# Bacterial respiration during stationary phase induces intracellular damage that leads to delayed regrowth

**DOI:** 10.1016/j.isci.2022.103765

**Published:** 2022-01-15

**Authors:** Spencer Cesar, Lisa Willis, Kerwyn Casey Huang

**Affiliations:** 1 Department of Microbiology and Immunology, Stanford University School of Medicine, Stanford, CA 94305, USA; 2 Department of Bioengineering, Stanford University, Stanford, CA 94305, USA; 3 Chan Zuckerberg Biohub, San Francisco, CA 94158, USA

**Keywords:** Microbiology, Cell biology, Functional aspects of cell biology, Mathematical biosciences

## Abstract

Bacterial survival is often challenged by nutrient-depleted conditions. Here, we show that *Escherichia coli* regrowth from prolonged stationary phase is heterogeneous. Some cells rejuvenated immediately, even after extended starvation, but others only restarted growth after a delay or not at all. The proportion of nongrowing cells increased with time spent in stationary phase, rather than time-dependent medium changes. Delayed regrowth was correlated with the dissolution of polar phase-bright foci likely representing damaged protein aggregates, and a deep learning algorithm distinguished cellular fates based on single images. Delayed regrowth initiated after upregulation of chaperones and DNA-repair enzymes, and deletion of a chaperone compromised stationary-phase morphology and increased the nongrowing cell proportion. Mathematical modeling of damage accumulation and division-mediated partitioning quantitatively predicted all rejuvenation statistics. Cells regrew immediately after starving in the absence of respiration. These findings reinforce the importance of intracellular damage control when nutrients are sparse, and repair when nutrients are plentiful.

## Highlights

• *Escherichia coli* rejuvenation from stationary phase is heterogeneous and age-dependent
• Delayed regrowth is correlated with dissolution of foci representing damaged proteins
• Modeling of damage accumulation and partitioning predicts rejuvenation statistics
• Delayed regrowth was dependent on the ability to respire in stationary phase

## Graphical abstract

**Figure:**
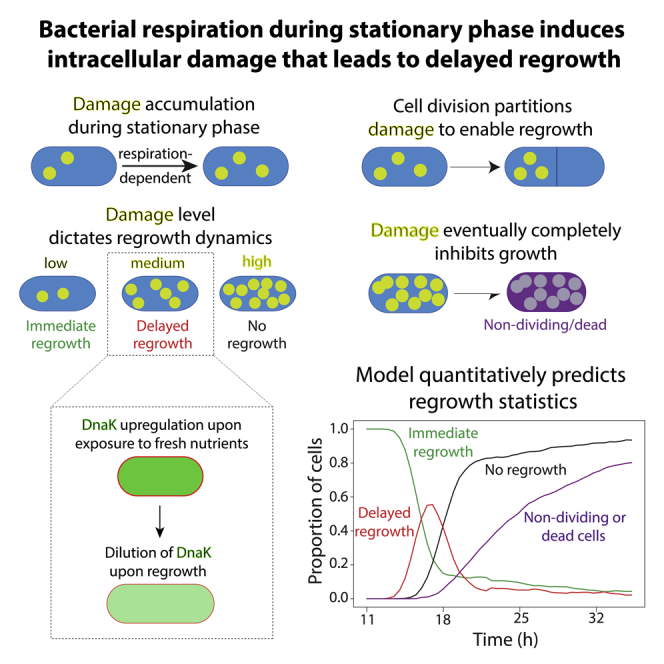

## Introduction

Many bacteria in the environment live in nutrient-sparse conditions, motivating a deeper understanding of the physiological effects of starvation. As cells grow, they deplete the environment of nutrients and build up waste products that alter the environment. *Escherichia coli* cells respond to these stressors through the sigma factor RpoS, which controls the expression of genes involved in acetate metabolism, pH homeostasis, and protection from oxidative stress during the initial stages of entry into stationary phase (Hengge-Aronis, 1993, 1999, 2002). Stationary-phase *E. coli* cells are typically thinner and shorter than log-phase cells because of successive reductive divisions upon entry into stationary phase (Kolter et al., 1993), and cells can remain metabolically active deep into stationary phase (Gefen et al., 2014), suggesting that stationary-phase cells are not fully depleted for nutrients and that cellular physiology adapts during starvation.

The limited nutrient availability during stationary phase means that bacteria face particular challenges in maintaining homeostasis and viability. For example, genetic perturbations to the lipid transport machinery do not affect exponential growth but cause outer membrane vesiculation and cell lysis in stationary phase (Sutterlin et al., 2016). Deletions of the superoxide dismutases in *E. coli* result in massive cell death during stationary phase after growth in aerobic conditions (Benov and Fridovich, 1995), highlighting the importance of controlling reactive oxygen species. Cells respond to starvation in a variety of manners, each of which can be informative about stationary phase. In rich media, after days in stationary phase, viability decreases by several orders of magnitude and a population of *rpoS* mutants emerges (Farrell and Finkel, 2003; Zambrano and Kolter, 1996), suggesting that at least some cells continue to grow. *rpoS* mutants can outcompete cells from earlier in stationary phase in starvation conditions (Farrell and Finkel, 2003; Zambrano and Kolter, 1996), but have lower fitness than wild-type cells after 24 h of regrowth in fresh medium (Zambrano et al., 1993), indicating tradeoffs between survival of stationary phase and rejuvenation.

The nutrient environment can greatly alter cellular survival in stationary phase and rejuvenation after starvation. During growth in minimal media, five days in stationary phase were required to reduce regrowth capacity and viability to levels comparable to LB after only one day in stationary phase (Luidalepp et al., 2011). A theoretical model that postulated the slow accumulation of growth-inhibiting complexes during stationary phase predicted that mean lag time should scale with the amount of time spent in stationary phase and that the lag-time distribution should have a long tail (Himeoka and Kaneko, 2017). Indeed, a recent study showed that within a single stationary-phase population of *E. coli* cells, the distribution of single-cell lag times upon dilution into fresh medium can be highly heterogeneous, with a power-law tail that extends from 1 h to >15 h (Simsek and Kim, 2019). In *Pseudomonas aeruginosa*, loss of multiple proteases accelerated cell death during growth arrest (Basta et al., 2020), indicating that active cellular processes maintain viability during starvation (Reeve et al., 1984).

In addition to delayed regrowth and death, some starved cells enter a viable but nonculturable state, defined by prolonged metabolic activity but absence of growth for long periods of time (in some cases years) in permissible conditions (Bergkessel et al., 2016). During aerobic growth, the nonculturable subpopulation of starved *E. coli* cells has more oxidative damage and higher levels of many genes and gene products related to oxidative stress (Desnues et al., 2003), including chaperones such as DnaK and GroEL that repair misfolded proteins in protein aggregates (Doyle et al., 2015; Kedzierska et al., 2001; Mogk et al., 1999). The DnaK and GroEL regulator RpoH (Jenkins et al., 1991) is upregulated as cells transition from stationary to exponential phase before the first cell division (Wagner et al., 2009), and transcriptional and posttranslational positive feedback between RpoH and DnaK/GroEL (Gamer et al., 1996) ensures high levels of chaperone expression when damaged proteins accumulate. However, how oxidative stress and chaperone production dictate the regrowth of single cells after starvation has yet to be determined.

Protein aggregates have been associated with growth-deficient bacterial cells in diverse contexts, including during steady-state growth, antibiotic persistence, and aging. After many generations of steady-state growth, aggregates accumulate at the older poles of aging *E. coli* cells, and are asymmetrically distributed after division (Lindner et al., 2008). Cells with aggregates had reduced reproductive ability relative to their sister cells without aggregates (Lindner et al., 2008), and new daughters had slightly faster growth rates than old daughters (Proenca et al., 2019). Persisters are a clinically relevant subpopulation of nongrowing cells defined by their survival of intense antibiotic treatment without resistance (Balaban et al., 2004, 2019). The formation of *E. coli* persisters in stationary phase was associated with protein aggregation (Leszczynska et al., 2013). After longer periods of time spent in stationary phase, persisters exhibit greater delays before initiating regrowth (Joers et al., 2010; Luidalepp et al., 2011), and lag time before resuscitation was related to recruitment of the chaperone DnaK (Pu et al., 2019) for facilitating protein disaggregation (Doyle et al., 2015). Inhibition of respiration in stationary phase dramatically reduced the formation rate of persisters, which was correlated with cellular redox activity (Orman and Brynildsen, 2015). Thus, aggregates appear to be a general sign of growth impairment in stressful conditions, but the links to lag time and redox activity in stationary phase remain unknown.

Here, we show that stationary-phase *E. coli* cultures develop into three distinct subpopulations based on their response to fresh nutrients: those that grow immediately, those with extended lag before regrowth (delayed regrowth), and those that do not resume growth at all. The fraction of delayed regrowth and nongrowing cells increased over time, whereas >5% of cells persisted in being able to grow immediately even after 36 h of incubation. We show that regrowth deficiency is because of intracellular damage rather than waste buildup in the medium and is correlated with the formation of large, polarly localized aggregates that dissipate upon the initiation of regrowth. Cells exhibiting delayed regrowth increased their expression of chaperones throughout lag phase, and deletion of the chaperone DnaK disrupted cell shape in stationary phase and reduced regrowth capacity. A minimal model of the accumulation of damage and its asymmetric partitioning via cell division in stationary phase was in quantitative agreement with our experimental measurements and thus further supports the notion of damage-induced growth impairment. A pulse of fresh medium during stationary phase was sufficient to postpone delayed regrowth, further indicating that fresh nutrients enable the dissolution of damage aggregates. Regrowth-deficient subpopulations did not develop during anaerobic stationary-phase incubation in the absence of nitrate, highlighting the impact of respiration on resuscitation.

## Results

### Culture age affects the heterogeneity of emergence from starvation

**Figure 1:**
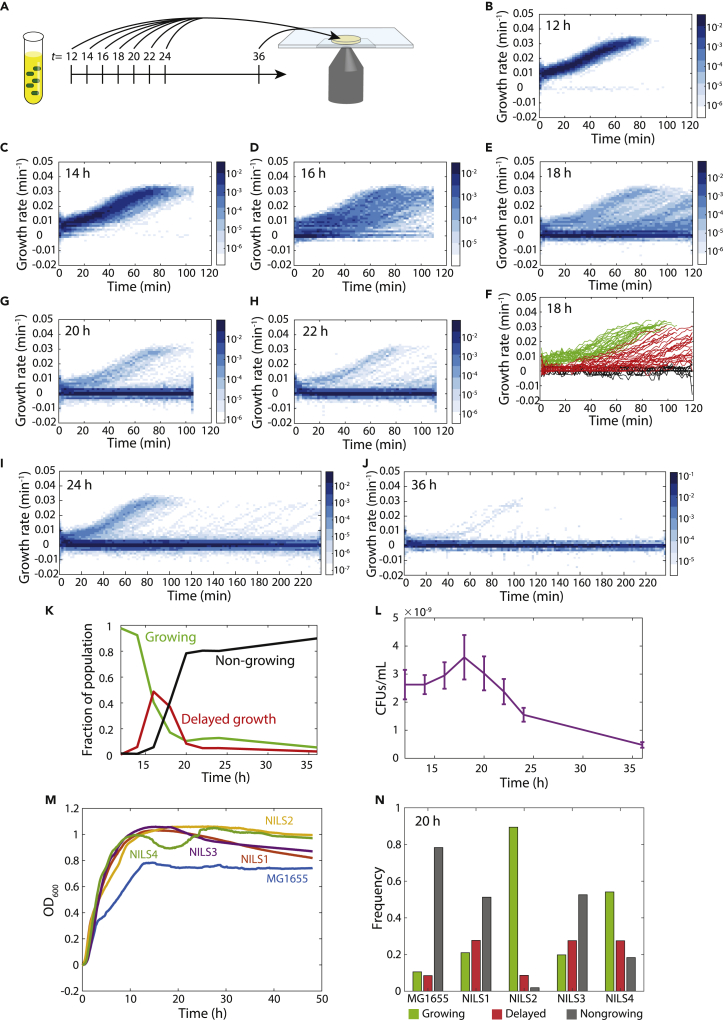
Increased time in stationary phase results in heterogeneous regrowth upon exposure to fresh medium (A) Schematic of protocol. Cells were sampled from a culture in stationary phase at the indicated times (in hours) and imaged on agarose pads with fresh LB to ascertain regrowth behaviors. (B–E and G–J) Heatmaps of the distribution of instantaneous growth rates over time for cells placed on agarose pads made with fresh LB after 12 h (B), 14 h (C), 16 h (D), 18 h (E), 20 h (G), 22 h (H), 24 h (I), and 36 h (J). Cell area was used to calculate growth rate using the formula 1/*A dA*/*dt*. As the time in stationary phase increased to 16–18 h, the distribution of growth rates broadened and an increasing number of cells were nongrowing. Past 18 h, the fraction of nongrowing cells continued to increase. *n* > 200 cells for each time point in every distribution.(F) Single-cell growth trajectories after 18 h in stationary phase, classified as immediately growing (green), delayed regrowth (red), and nongrowing (black) (STAR Methods). *n* > 200 cells. (K) The fraction of immediately growing cells began to decrease at 14 h but remained at 5–10% even after 36 h. The delayed-growth fraction peaked at 16 h and then decreased below the fraction of immediately growing cells. The nongrowing fraction became the majority at ∼18 h. All fractions were computed from *n* > 200 cells. (L) The number of colony forming units (CFUs) increased slightly from 12 h to 18 h, suggesting a slow rate of division, and then steadily decreased thereafter. *n* = 3 technical replicates, error bars represent 1 standard deviation (SD). (M) Four *E. coli* natural isolates all displayed higher yields than MG1655. Growth curves are means of *n* = 3 technical replicates. (N) After 20 h, MG1655 and the natural isolates exhibited distinct fractions of immediately growing, delayed-growth, and nongrowing cells. All fractions were computed from *n* > 200 cells.

Previous work showed that starvation can generate persisters, and longer periods in stationary phase led to an increase in the delay before regrowth (Joers et al., 2010; Luidalepp et al., 2011). To reproducibly quantify when and to what extent individual cells enter a state of delayed or inhibited growth during stationary phase, we established a reproducible protocol in which we inoculated a culture using log-phase *E. coli* cells at OD_600_ = 0.1 (STAR Methods). Starting at 12 h after inoculation, we extracted 1 μL of *E. coli* MG1655 cells every 2 h until 36 h, spotted them onto a 1% agarose LB pad, and acquired multi-hour time-lapse movies while incubating at 37°C. To determine regrowth dynamics, we segmented trajectories of hundreds of cells from each movie and computed the instantaneous growth rate over time (Figure 1A, STAR Methods).

At 12 h, virtually all cells (>98%) resumed growth immediately and homogeneously, accelerating to their maximum growth rate of ∼0.03 min^−1^ (doubling time of ∼20 min) after ∼60 min (Figure 1B). This acceleration was markedly slower than exponential-phase cells, which reached a growth rate of ∼0.03 min^−1^ after only a few minutes as expected, because they were already adapted for rapid growth (Figure S1A). By 14 h, some cells exhibited a longer delay in growth (Figure 1C). By 16 h, the fraction of cells with delayed regrowth increased, and a small population (∼5%) that did not grow for the entire period of imaging (henceforth referred to to as “nongrowing”) emerged (Figure 1D), potentially similar to the heterogeneity of persister cell regrowth (Pu et al., 2019). Cells with delayed regrowth typically had similar acceleration trajectories as the immediately growing cells once they began growth (Figure S1B). At 18 h, the majority of cells did not resume growth while imaging (Figure 1E). We identified thresholds based on the initial and final growth rates of each cell that robustly separated the three groups (immediate growth, delayed regrowth, and nongrowing) for all culture ages (STAR Methods, Figure 1F). As the age of the culture increased, cells with delayed regrowth exhibited longer delays (lag time) and increased heterogeneity (Figures S1C–S1H), and the fraction of nongrowing cells increased (Figures 1E,1G–1J). In addition, among the growing cells the initial growth rate upon exposure to fresh medium decreased with culture age (Figure S1I). Nonetheless, cultures older than 24 h still maintained a small population of cells that grew immediately (Figures 1I and 1J), suggesting they had not suffered ill effects of starvation.

Quantification of the prevalence of each subpopulation across culturing time revealed a transition from predominantly immediate growth at 12 h to a mixture of the three groups in which the delayed-growth fraction peaked at 16 h and then decreased with culture age (Figure 1K). Notably, although the nongrowing fraction dominated after 20 h, the immediate-growth fraction was larger than the delayed-growth fraction beyond this point and continued to account for >5% even up to 36 h (Figure 1K).

To determine whether the nongrowing cells were dead, we plated cultures at each time point and counted colonies. CFUs/mL increased slightly from 12 to 18 h, suggesting the occurrence of cell division in stationary phase (Figure 1L). After 18 h, CFUs/mL decreased steadily with culture age, with a ∼2-fold and ∼5-fold decrease relative to 12 h after 24 and 36 h, respectively (Figure 1L)). Overall, extended incubation in stationary phase induced heterogeneity in cellular recovery, including increased lag, and eventually a transition to nongrowth and cell death.

### Natural isolates exhibit variable fractions of growth-deficient cells

Laboratory strains such as MG1655 have been passaged in aerobic conditions for many generations, leading us to wonder if natural isolates of *E. coli* that generally reside in anaerobic environments within hosts would exhibit delayed regrowth. We selected four strains (NILS1-4) from a collection of *E. coli* clinical samples (Galardini et al., 2017) and grew them along with MG1655 for 20 h, the incubation time for which MG1655 first exhibited a high level of nongrowing cells (Figure 1K). Somewhat surprisingly, all four natural isolates grew to a higher maximum OD than MG1655 (Figure 1M). All four natural isolates exhibited delayed regrowth, but with distinct fractions of each population. Interestingly, for the same time spent in stationary phase, all four natural isolates had fewer nongrowing cells than MG1655 (Figure 1K). NILS2 had the highest fraction of immediately growing cells (>80%), whereas NILS3 had the lowest (<20%) (Figure 1N). These data indicate that delayed regrowth is generally conserved in *E. coli*, although with distinct kinetics across strains.

### Development of regrowth deficiency is driven by time spent in stationary phase and not changes in the extracellular environment

We considered two potential mechanisms for the cause of regrowth deficiency (in both the delayed regrowth and nongrowing populations): intracellular accumulation of damage or toxins, or modification of the extracellular environment based on production of secreted toxic by-products that affect cells directly or by modifying the environment. To distinguish between these two possibilities, we determined whether regrowth is driven by the time cells spent in culture or by the age of the stationary-phase supernatant. We inoculated cultures 2 h apart, so that the ages of the two cultures were different.

**Figure 2:**
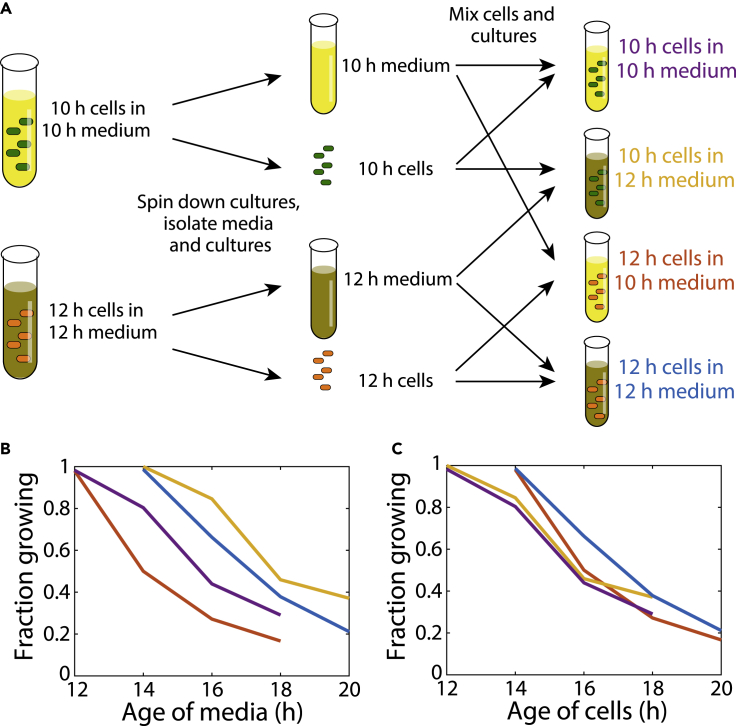
Delayed regrowth results from changes to intracellular physiology rather than the extracellular environment (A) Scheme to distinguish the effects of time spent in stationary phase compared with modification of the extracellular environment. Overnight cultures diluted into fresh LB were grown for 10 and 12 h. Each was spun down and the supernatant was separated from the cells, whereupon all pairwise combinations of supernatant and cells were constructed and incubation of the four cultures continued. Samples were taken every 2 h thereafter and the fraction of immediately growing cells on agarose pads with fresh LB was measured using time-lapse microscopy. (B) The age of the culture supernatant does not dictate the onset of regrowth deficiency. Cells from a 12-h culture resuspended in 10-h supernatant exhibited delayed regrowth ∼2 h later, whereas cells from an 10-h culture resuspended in 12-h supernatant exhibited delayed regrowth ∼6 h later. All fractions were computed from *n* > 200 cells. (C) The dynamics of the onset of delayed regrowth were highly similar for all four cultures in (B) when measured as a function of the time cells had been incubated. All fractions were computed from *n* > 200 cells.

Once the culture ages had reached 10 and 12 h, we spun down each culture to separate cells from their supernatant (STAR Methods). We selected these ages so that cells were in a state before the transition to dormancy, with the 2 h difference sufficient to cause a large difference in dormancy onset kinetics (Figure 1K). We then resuspended both pellets in each of the supernatants of the 10-h (“young”) and 12-h (“old”) cultures and continued to incubate the resuspended cultures (Figure 2A). Samples were extracted for imaging every 2 h thereafter, and we quantified the fractions of cells that were able to grow immediately.

The fraction of growing cells as a function of the time elapsed since the medium in each mixture was inoculated exhibited substantial variability, with the time at which the majority of the population became growth-deficient ranging from ∼14 to 18 h (Figure 2B). By contrast, the growing fraction as a function of the culturing time was similar across mixtures (Figure 2C), with the majority of cells becoming growth-deficient at ∼16 h regardless of whether they were resuspended in young or old supernatant. These data indicate that it is the age of the cell population and not the age of the supernatant that drives the loss of the ability of cells to immediately resume growth when exposed to fresh nutrients, and that growth deficiency is a result of accumulated physiological changes within cells.

### Visible protein aggregates form in stationary phase and dissolve during regrowth

**Figure 3:**
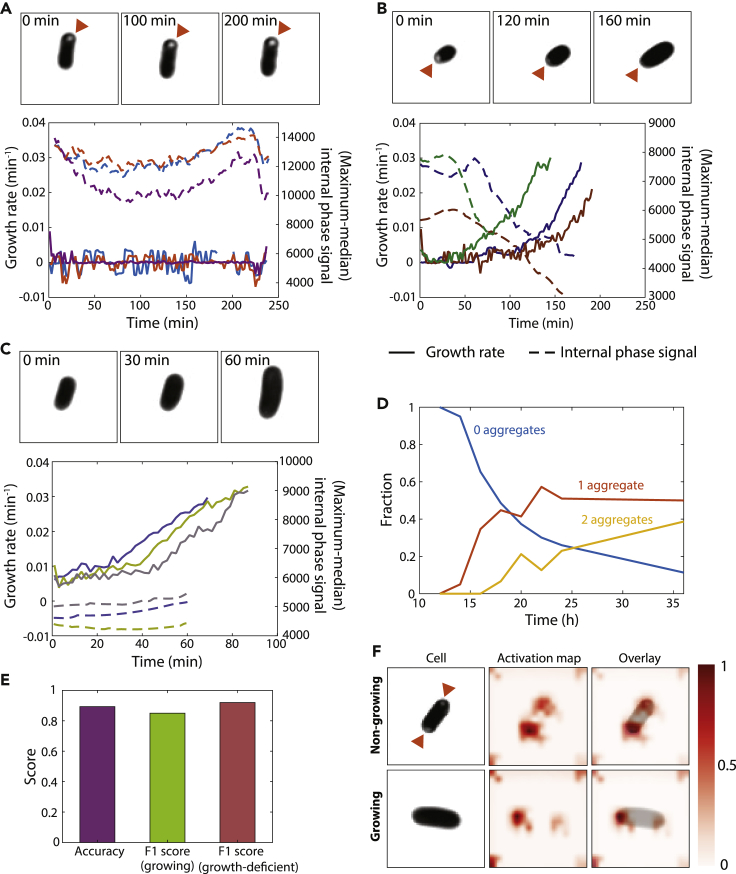
Delayed regrowth is associated with the dissolution of bright cytoplasmic foci that develop in stationary phase (A) Cells from a 20-h culture often exhibited visually apparent bright cytoplasmic foci (arrowheads) that were almost always located near one of the poles. Top: phase-bright foci persisted in nongrowing cells. Bottom: the difference between the maximum and median phase signal inside the cell over time was used as an indicator of foci, and the intensities of foci in three representative cells remained approximately constant over time (similar behavior was observed in *n* > 40 cells). Solid lines are growth rates and dashed lines are the difference between the maximum and median phase signal. Phase-contrast images were processed by adjusting the brightness and contrast to emphasize the phase-bright foci. (B) Phase-bright foci dissolved from delayed-growth cells, coincident with the restart of growth, suggesting that the foci are connected with growth inhibition. Top: images were processed as in (A). Bottom: the intensities of foci in three representative cells started to decrease coincident with the growth rate increasing from zero (similar behavior was observed in *n* > 40 cells). Solid lines are growth rates and dashed lines are the difference between the maximum and median phase signal. (C) Phase-bright foci were absent from immediately growing cells. Top: images were processed as in (A). Bottom: the intensities of foci in three representative cells remained low over time (similar behavior was observed in *n* > 40 cells). Solid lines are growth rates and dashed lines are the difference between the maximum and median phase signal. (D) The number of cells with foci increased over time, and cells transitioned from predominantly having zero foci to one or two foci similar to the transition to delayed regrowth and nongrowing cells (Figure 1K). All fractions were computed from *n* > 200 cells. (E) A deep learning algorithm trained on phase-contrast images of cells from various cultures ages was able to accurately classify cells as immediately growing or growth-deficient (delayed/nongrowing). The F1 score is the harmonic mean of precision and recall. (F) Activation maps demonstrate that the algorithm in (E) used information from the polar regions overlapping with foci to classify cells.

We noticed that cells from 18-h cultures, which had a high proportion of delayed and nongrowing cells (Figure 1E), often had bright cytoplasmic foci visible in phase-contrast images (Figures 3A and 3B). Previous studies of aging *E. coli* cells identified polar punctae of a YFP fusion to the small heat shock protein IbpA, which binds and colocalizes with protein aggregates (Lindner et al., 2008). Other studies observed aggregates associated with antibiotic persistence and dormant cells that dissipated if and when growth resumed (Pu et al., 2019; Yu et al., 2019). Thus, we hypothesized that the foci in stationary-phase cells represented aggregates that prevented immediate regrowth. Indeed, phase-bright foci persisted in nongrowing cells (Figure 3A), dissolved from delayed-growth cells when growth restarted (Figure 3B), and were completely absent from all immediately growing cells (Figure 3C). The foci were almost always located at poles and became more prevalent as the culture aged (Figure 3D); the increase in the number of cells with one and two foci coincided with the increases in both the delayed regrowth and nongrowing fractions (Figure 1K) and the drop in viability (Figure 1L), respectively. These data support previous findings that aggregates are strongly associated with regrowth deficiency.

We trained a deep learning algorithm on the first frame of phase-contrast time-lapse movies from cultures of various ages. For all movies, we used subsequent frames to classify cells as immediately growing or regrowth-deficient (delayed/nongrowing) as before. Our algorithm was able to accurately classify ∼90% of cells (none of which were used for algorithm training) with an F1 score of 0.8–0.9 (Figure 3E), indicating that the future growth state of most cells is identifiable at the time of exposure to new nutrients. Activation maps showed that the classification algorithm focused on the polar regions of cells (Figure 3F), consistent with the location of aggregates. These findings demonstrate that the appearance of cells can be used to predict future behavior once they are shifted to high nutrient conditions, and suggest that aggregates are a general signature of growth inhibition during starvation.

### Cells that take longer to emerge from stationary phase are smaller in size

Because aggregates were quantized and polarly localized, we speculated that the concentration of aggregates might be affected by cell size. A previous study showed that cell size in stationary phase was strongly correlated with the capacity for growth and that smaller cells were more likely to be persisters (Bakshi et al., 2021). We previously discovered that lag time during emergence from stationary phase for a *mreB*^*A53T*^ mutant with larger average cell width and volume was lower in glucose-supplemented minimal medium (Monds et al., 2014). To determine if regrowth was dependent on cell size in stationary-phase cultures in LB, we computed the initial cell sizes of each cell (STAR Methods) from the first frame of a time-lapse movie of a 20-h culture with a large fraction of nongrowing cells. We found that cells with delayed regrowth were on average smaller than immediately growing cells, and nongrowing cells were yet smaller (Figure S2A), consistent with previous studies (Bakshi et al., 2021).

To determine whether alteration of cell size via mutation was sufficient to alter regrowth capacity, we performed similar experiments with an MG1655 *mreB*^*A53T*^ mutant that has a larger volume than wild-type cells (Figures S2A–S2C). In 20-h cultures, we found the same qualitative relationship between cell size and regrowth behavior for the mutant as for wild-type (Figure S2C). Moreover, there was a significantly larger fraction of immediately growing *mreB*^*A53T*^ cells and a smaller fraction of nongrowing cells than in a wild-type culture (Figure S2D), and the initial growth rates of immediately growing *mreB*^*A53T*^ cells were higher than those of wild-type cells (Figure S2E). Thus, cell size is associated with the ability of cells to emerge from stationary phase. However, the mean initial size of nongrowing *mreB*^*A53T*^ cells was larger than that of immediately growing wild-type cells, indicating that a threshold size does not dictate regrowth behavior.

### Cells with delayed regrowth increase expression of chaperones before resuscitation

A previous study suggested that nonculturable starved cells exhibit higher levels of several damage-associated stress regulons, including the transcripts *groEL* and *sodC* and the proteins RpoS and DnaK (Desnues et al., 2003; Pu et al., 2019), and another study demonstrated that DnaK colocalized with aggregates and was critical for dissolving aggregates (Doyle et al., 2015) and resuming growth (Pu et al., 2019). To investigate the expression of these damage-associated regulons in cells with delayed regrowth from stationary phase, we performed experiments with *E. coli* MG1655 strains containing a GFP-reporter expressed from the promoter of candidate genes (here, these reporter strains are referred to to as *P*<*gene*>:*gfp*) (Zaslaver et al., 2006). We first focused on *dnaK*, which encodes a chaperone involved in DNA replication and dissolution of protein aggregates (Kedzierska et al., 2001; Mogk et al., 1999).

**Figure 4:**
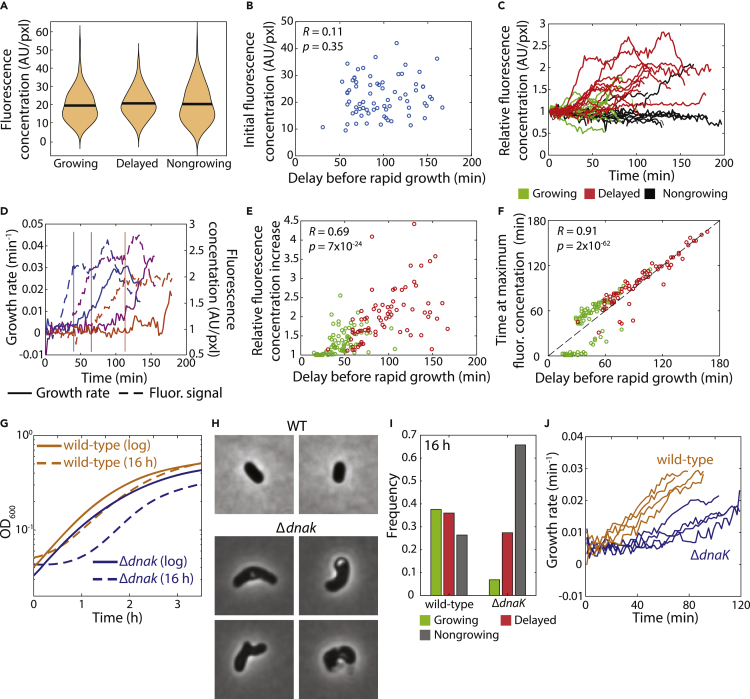
Chaperone activity is important for the resuscitation of regrowth-deficient cells and for survival of stationary phase (A) Initial fluorescence concentration in stationary-phase cells with a *gfp* reporter expressed from the *dnaK* promoter was similar for cells in all regrowth groupings. (B) Among delayed-growth cells, the initial GFP concentration was uncorrelated with the delay before rapid growth (>0.02 min^−1^), indicating that *dnaK* expression in stationary phase is not a predictor of regrowth behavior. Student’s t-test, *n* = 72 cells. (C) In delayed-growth cells (red), GFP concentration (normalized by initial fluorescence) increased, whereas in immediately growing (green) or nongrowing (black) cells, intensity remained approximately constant over time. *n* = 31 cells. (D) In delayed-growth cells (three representative examples shown here, similar behavior was observed in *n* > 40 cells), GFP concentration (dashed curves) (normalized by initial fluorescence) increased up to a plateau before the instantaneous growth rate (solid curves) started to increase from zero. Dashed and solid curves of the same color indicate the same cell. (E) In delayed-growth and immediately growing cells, the maximum increase in GFP concentration relative to the initial value from stationary phase was highly correlated with the delay before rapid growth. Student’s t-test, *n* > 100 cells. (F) For most of the cells in (E), the time at which the maximum fluorescence was reached was approximately coincident with the delay before rapid growth was achieved. Dashed line is *y* = *x*. Student’s t-test, *n* > 100 cells. (G) Growth curves of BW25113 wild-type and Δ*dnaK* cultures started from log phase were similar, while the Δ*dnaK* growth curve started from stationary phase exhibited increased lag relative to wild type. Growth curves are means of three technical replicates. (H) Δ*dnaK* cells from a 16-h culture exhibited aberrant morphologies compared with the short rods typical of a wild-type culture. (I) A significantly higher fraction of Δ*dnaK* cells compared with wild-type cells were nongrowing after 16 h, and very few grew immediately. Fractions were computed from *n* > 100 cells. (J) Of the small population of immediately growing Δ*dnaK* cells, the initial growth rate was significantly lower than wild-type cells. Shown are a representative subset of four cells of each genotype; similar behavior was observed in *n* > 10 cells of each genotype.

Time-lapse imaging of *PdnaK*:*gfp* cells from a 20-h culture on agarose with fresh LB revealed subpopulations of delayed-growth and nongrowing cells, as we observed for wild-type. Initial GFP concentrations were similar for immediately growing, delayed-growth, and nongrowing cells (Figure 4A), and were uncorrelated with the time delay before rapid growth (Figure 4B). Thus, stationary-phase *dnaK* expression is not predictive of regrowth delay. In immediately growing or nongrowing *PdnaK*:*gfp* cells, GFP fluorescence concentration remained approximately constant throughout imaging (Figure 4C). Interestingly, in cells with delayed regrowth, GFP fluorescence concentration increased substantially, indicating that expression of DnaK was specifically induced in these cells but only after the shift to fresh medium (Figure 4C). Growth rate typically increased after an initial rise and plateau of *dnaK* expression (Figure 4D), the fold-increase in *dnaK* expression was proportional to the delay before rapid growth (Figure 4E), and in all but cells with the shortest delays the maximum in fluorescence concentration occurred at approximately the same time as growth resumption (Figure 4F). These data suggest that cells with longer lag times had higher levels of damage and hence required more chaperone activity.

We observed behavior quantitatively similar to DnaK in *PgroE*:*gfp* (GroEL is a chaperone) cells (Figure S3A), further supporting the notion that delayed regrowth is because of accumulated protein damage. By contrast, for several promoters not related to protein damage including *recA* (which encodes a DNA repair enzyme), *ompA* (an abundant outer membrane porin), *pyk* (pyruvate kinase), *tnaC* (tryptophan operon leader peptide), and *tufA* (translation elongation factor Tu 1), GFP concentration increases were not strongly correlated with the delay until rapid growth (Figures S3B–S3F), arguing against the need to boot up metabolic, translational, or cell-envelope synthesis capacity. Interestingly, *PrpsB*:GFP showed a slightly negative correlation with the delay before regrowth, suggesting that some processes necessary for growth are specifically downregulated in delayed regrowth cells (Figure S3G).

To determine whether DnaK is critical for regrowth from stationary phase, we examined the Δ*dnaK* knockout from the Keio collection (Baba et al., 2006) and its parent strain BW25113. Growth curves of BW25113 wild-type and Δ*dnaK* cultures, which were inoculated using log-phase cells at an OD_600_ = 0.1, were similar (Figure 4G), indicating that the mutant can grow comparably to wild-type in log phase. However, Δ*dnaK* cultures inoculated from stationary phase exhibited longer lag than wild-type (Figure 4G), suggesting that growth defects emerge in stationary phase. Indeed, after 16 h of incubation, Δ*dnaK* cells exhibited distorted morphologies (Figure 4H) in stark contrast with the typical short rods of wild-type cells. Whereas most wild-type BW25113 cells regrew immediately or exhibited delayed regrowth, ∼70% of Δ*dnaK* cells were nongrowing (Figure 4I). Of the small subpopulation of immediately growing cells, their growth rate kinetics were substantially slower than the immediately growing population of wild-type cells (Figure 4J). Moreover, 15–20% exhibited a range of growth-impaired behaviors not observed in wild-type cells, including growth for only a short period upon transition to fresh LB as well as cycles of expansion and shrinking (Video S1). These data confirm the pivotal role of DnaK in surviving stationary phase and in resuming growth in fresh medium, presumably by dissolving damage aggregates that accumulate during starvation.

Video S1. Some Δ*dnaK* cells exhibit cycles of growth and shrinking during emergence from stationary phase, related to Figure 4 Upper left: the time since being exposed to fresh medium, in hours and minutes

### Mathematical model quantitatively predicts the kinetics of delayed regrowth

Our imaging strongly suggested a link between regrowth of cells with delayed regrowth and the dissolution of phase-bright foci (Figure 3), but so far causality has not been established. Thus, we sought to test whether our findings are quantitatively consistent with a model of damage-mediated growth inhibition. The increased fraction of nongrowing cells in older cultures (Figure 1K) suggested that damage is ongoing throughout stationary phase. Because aggregates appeared primarily at the poles (Figures 3A and 3B), we surmised that division of a cell with only one aggregate would segregate most damage to a single daughter cell, restoring the other daughter to a (nearly) undamaged state, similar to previous findings of the rejuvenation of older cells via asymmetric aggregate segregation after division (Lindner et al., 2008). The increase in CFUs/mL from 12 to 18 h (Figure 1L) suggested that at least some cells are capable of continued growth and/or division in stationary phase, albeit slowly because of nutrient limitation.

**Figure 5:**
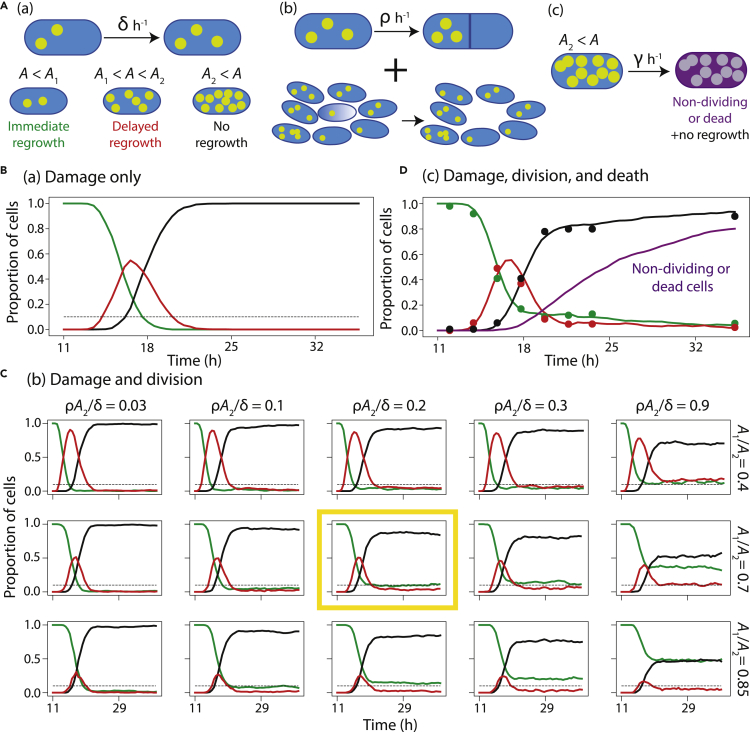
A model of stationary-phase damage accumulation recapitulates the kinetics of delayed growth onset when lag time is damage-dependent and a subset of cells divide slowly. (A) (a) In a model with only damage, throughout stationary phase starting ∼11 h after inoculation, damage *A* accumulates at rate δ h^−1^. Upon restoration to fresh medium, cells grow immediately if *A* ≤ *A*_1_, grow with a delay if *A*_1_ < *A* ≤ *A*_2_, and do not grow during the period of observation if *A* > *A*_2_. (b) Division is incorporated at rate ρ h^−1^. Upon division, damage partitioning is asymmetric such that one daughter receives all the damage. (c) Cells with *A* > *A*_2_ stop dividing or die (without lysis) at rate *γ* h^−1^, such that they do not form colonies upon restoration to fresh medium during CFU counting. (B) Simulations based on model (a) with damage only using $A_{1}/A_{2}\approx 0.7$ and $A_{2}\approx 20$ recapitulated the early fractions of immediately growing (green), delayed (red), and nongrowing (black) subpopulations. However, the persistent subpopulations with immediate and delayed regrowth after ∼20 h did not occur. The horizontal dashed line represents the experimental estimate of the fraction of immediately regrowing cells at 24 h of 0.1. (C) The addition of division with asymmetric-damage partitioning (model (b)) with $A_{1}/A_{2}\approx 0.7$, $\rho A_{1}/\delta \approx 0.2$, and *A*_2_ = 20 recapitulated the early fractions and the long-term persistent subpopulations of immediate and delayed regrowth (yellow box). Deviations from these parameter values altered the dynamics. (D) Simulations in which cells become nonculturable as damage accumulates (model (c)) produced excellent fits to experimental data (circles, Figure 1K) for $\gamma A_{1}/\delta =0.77$, $\rho A_{1}/\delta =0.15$, $A_{1}/A_{2}=0.7$, and *A*_2_ = 25. By 36 h after inoculation, ∼80% of cells were predicted to be nonculturable, consistent with CFU measurements (Figure 1L).

In our model, we assume that damage accumulates spontaneously throughout stationary phase and high levels of damage cause a delay in regrowth and eventually lead to a nongrowing state. Damage accumulates from 0 upon entry into stationary phase (which we estimate at ∼11 h after inoculation) probabilistically at a rate δ. Cells with damage < *A*_1_ grow immediately upon restoration to fresh medium, cells with damage between *A*_1_ and *A*_2_ experience delayed regrowth, and cells with damage > *A*_2_ are assumed to be nongrowing (at least during the period of observation; Figure 5A). Simulations showed that cells transit from immediate to delayed regrowth on average at time *A*_1_ / *δ* and then to nongrowth at *A*_2_ / *δ*, with a peak in the fraction of delayed-growth cells of magnitude ∼ $\sqrt{A_{2}}\left(1-A_{1}/A_{2}\right)/\sqrt{1+A_{1}/A_{2}}$ (Figure 5B). For *A*_1_ / *δ* = 5 h, *A*_2_ / *δ* = 7 h, and *A*_2_ = 20, the model recapitulated regrowth statistics up to, but not beyond, ∼20 h after inoculation (Figures 5B and S4A).

Without the potential for cellular recovery, the model predicts a negligible proportion of immediately growing cells after ∼20 h (Figure 5B), coincident with the transition to nongrowth (because cells only become progressively more damaged), which is inconsistent with the observed persistence of an immediately growing population (Figure 1K). Thus, we incorporated into our model division at a probabilistic rate ρ. Division restores one daughter cell into an undamaged (and thus immediately growing) state, whereas the other daughter inherits all damage (Figure 5A). At long times, the proportion of immediately growing cells is $\frac{\frac{\rho A_{1}}{\delta }}{1+\frac{\rho A_{1}}{\delta }}$ and the ratio of immediately growing to delayed regrowth cells is $\frac{\rho A_{1}}{\delta }+\frac{\frac{A_{1}}{A_{2}}}{1-\frac{A_{1}}{A_{2}}}$ (Figure S4B). Our experimental measurements indicate that 36 h after inoculation (Figure 1K), $\frac{\frac{\rho A_{1}}{\delta }}{1+\frac{\rho A_{1}}{\delta }}\approx 0.1$ and $\frac{\rho A_{1}}{\delta }+\frac{\frac{A_{1}}{A_{2}}}{1-\frac{A_{1}}{A_{2}}}\approx 2-3$, hence $\rho A_{1}/\delta \approx 0.11$, $A_{1}/A_{2}\approx 0.7$, and the average number of divisions it takes a cell to reach a nongrowing state is $\rho A_{2}/\delta \approx 0.16$. Remarkably, simulations with these parameter values (Figure 5C) recapitulated both early and late stationary-phase regrowth statistics (Figure 1K), whereas significant deviations from these values resulted in poor fits to the data (Figure 5C), regardless of whether absolute levels or concentrations of intracellular damage trigger delayed regrowth (Figure S4C). The predicted time between divisions is 1ρ≈A1δ0.2−A1δ0.1≈25−50h. Results were similar if the division rate declines linearly with increased damage (SI, Figure S4D), which is plausible because FtsZ is sequestered in the aggregates that form at the cell poles in stationary phase (Figure S5) (Yu et al., 2019).

Viability declined starting ∼18 h after inoculation (Figure 1L), approximately coincident with the transition to nongrowth. We assessed the ability of our model (Figure 5A) to account for the number of cells in a nonviable state late in stationary phase by including a transition to nonviability at a rate *γ* after accumulating *A*_2_ of damage. For $\gamma A_{1}/\delta \approx 0.8$, $\rho A_{1}/\delta \approx 0.18$, and $A_{1}/A_{2}\approx 0.65$, our model predicted that ∼80% of cells become nonviable by 36 h (Figure 5D), similar to our experimental data (Figure 1L). In sum, a minimal model containing reasonable assumptions quantitatively explains the trajectories of immediately growing, delayed-growth, nongrowing, and nonviable cells as a function of culture age, suggesting that a single variable that accumulates over time (i.e., damage) and is asymmetrically partitioned by cell division is sufficient to explain the kinetics of delayed regrowth.

### A pulse of fresh medium delays the development of growth deficiency

To confirm if the intracellular damage built up during starvation is reversible, we administered a pulse of fresh medium at a time point just before cells were entering a delayed regrowth state and quantified its effects on regrowth kinetics. We grew dilutions of two overnight cultures for 12 h, and then subjected the cultures to separate treatments. One was spun down and resuspended in twice its volume of fresh medium. The second was spun down and resuspended in its own spent supernatant.

We incubated both cultures for 20 min, resuspended them in spent LB from a 12 h-old culture, and incubated them for a further 10 h. Although it is possible that a small subset of cells underwent cell division during the 20-min period, we previously showed that essentially no cell divisions occur for the first hour when *E. coli* cells emerge from stationary phase in the presence of fresh LB (Shi et al., 2021). We extracted samples every 2 h, and imaged cells on agarose pads with fresh LB to examine their emergence from stationary phase. For both cultures, most cells were able to grow immediately at 12 h (Figure S6). The second culture (no pulse of fresh medium) behaved as before, with a gradual increase in the fraction of growth-deficient cells such that ∼50% were growth deficient 4–6 h later (Figure S6). By contrast, the 20-min pulse of excess fresh medium was sufficient to ensure that after 4 h back in spent medium almost all cells retained the ability to resume growth immediately on fresh medium (Figure S6). Nonetheless, a similar fraction of cells were growth-deficient (∼80%) in all cultures after 10 h (Figure 2D), suggesting that the pulse reversed or delayed damage but only temporarily. The observation that the pulse had an effect even though it was administered before any cells displayed delayed regrowth is consistent with accumulation of damage before detectable growth impairment.

### Cells do not exhibit delayed regrowth in the absence of respiration

Inhibition of respiration in stationary phase reduces the formation of persisters (Orman and Brynildsen, 2015), and protein aggregation during stationary phase is oxygen-dependent (Kwiatkowska et al., 2008). In addition, oxidative damage has been shown to be higher in nongrowing versus growing cells (Desnues et al., 2003) and increasing redox stress accelerates the formation of growth-deficient cells (Pu et al., 2019). Thus, we hypothesized that the presence of oxygen during stationary phase leads to growth defects during rejuvenation. To test this hypothesis, we grew a culture in non-shaking conditions, which allows cells to rapidly deplete oxygen (Arjes et al., 2020). Now, after 20 h, most cells were able to grow immediately upon exposure to fresh medium (Figures S7A–S7C), unlike in shaking conditions.

To determine whether growth without oxygen prevents growth deficiency, we grew MG1655 cells in an anaerobic chamber using prereduced LB (STAR Methods). Periodically, a sample of cells was extracted for imaging in an aerobic environment. We observed a stark contrast with cells grown aerobically: in the anaerobic chamber, even 40 h after inoculation, all cells immediately resumed growth on fresh LB (Figure S7D), suggesting that respiration was the cause of delayed regrowth.

**Figure 6:**
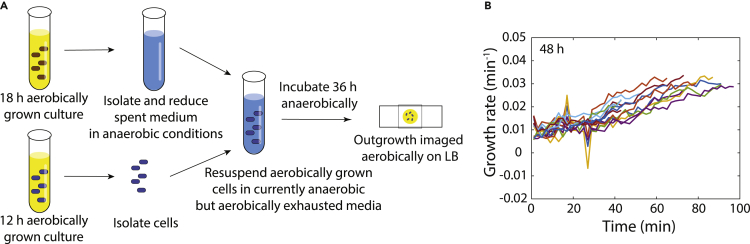
Anaerobic incubation in stationary phase prevents delayed regrowth. (A) A 12-h aerobically grown culture entering stationary phase was resuspended in the supernatant from an 18-h aerobically grown culture prereduced in an anaerobic chamber. The resulting culture was incubated in an anaerobic chamber for 36 h, and then outgrowth was monitored aerobically on fresh LB agarose pads. (B) After the protocol in (A), all cells grew immediately and accelerated in growth similar to aerobically grown cultures after 12 h of incubation (Figure 1B). Fifteen representative cells are shown (similar behavior was observed in *n* > 100 cells).

To test whether oxygen exposure specifically during stationary phase was responsible for delayed regrowth and to control for the different yields (Figure S7E) and extracellular environments of *E. coli* cultures grown aerobically and anaerobically, we grew a culture aerobically for 12 h and then resuspended it in supernatant from an 18-h aerobic culture (which had reached stationary phase) that had been prereduced in the anaerobic chamber (Figure 6A, STAR Methods). We then incubated the resuspended culture in the anaerobic chamber for a further 36 h. Similar to our observations involving cultures grown entirely within the anaerobic chamber (Figure S7D), all cells grew immediately (Figure 6B). Moreover, no cells (of >100 examined) displayed any sign of aggregates, suggesting that damage was minimal (or nonexistent). Finally, to determine if it was the absence of oxygen or the incapacity to respire that protected cells during anaerobic growth in LB, we grew cells anaerobically in LB+10 mM nitrate to allow for anaerobic respiration. Now, delayed and nongrowing populations emerged after 48 h, with only ∼40% of cells able to immediately resume growth (Figure S7F). These findings suggest that respiration during stationary phase is a primary cause of delayed regrowth, consistent with previous studies showing that oxidative damage increases growth deficiencies.

## Discussion

Here, we demonstrated that dilution of an aerobically grown stationary-phase *E. coli* culture into fresh medium led to a heterogeneous population of immediately growing, delayed-growth, and nongrowing cells (Figure 1). Longer times spent in stationary phase led to an increased fraction of nongrowing cells, although a small fraction remained able to grow immediately (Figure 1K). Cells with delayed regrowth exhibited bright foci that dissolved before the resumption of growth (Figure 3B) and after the synthesis of repair enzymes (Figures 4C–4F and S3A), supporting previous studies (Pu et al., 2019) that delayed regrowth is because of intracellular protein damage. When cells were grown in conditions that precluded respiration, cells did not exhibit growth deficiencies (Figures 6B and S7D) and bright foci could not be detected. Mathematical modeling based on gradual damage accumulation and division-mediated recovery through asymmetric partitioning of damage quantitatively predicted the fraction of each of the three subpopulations over time (Figure 5D), with maintenance of an immediately growing fraction of ∼10%. Although our model does not explicitly predict the dynamics of growth rate during resuscitation, our observation that initial growth declines with the amount of time spent in stationary phase (Figure S1H) is consistent with damage also lowering initial growth rate and with our observation that wider *mreB*^*A53T*^ cells exhibited both a larger fraction of growing cells and a higher initial growth rate (Figure S2E). Together, our evidence indicates that delayed regrowth is driven by the accumulation of damage generated in conditions that permit respiration (Figure S7F). This model should prove powerful for interpreting the molecular basis of delayed regrowth in other strains and species.

The ubiquity of delayed regrowth across natural isolates (Figures 1M and 1N) raises the question of whether delayed regrowth is an inevitable effect of damage or a selected response. Long lag times are beneficial for survival of antibiotic treatment (Balaban et al., 2004) and likely other stresses, despite their obvious disadvantages in conditions that permit rapid growth. Moreover, it is possible to select for mutants with increased lag and increased persistence (Kaspy et al., 2013), underscoring the genetic potential for increasing lag under certain selective pressures. Evolution experiments with repeated passaging have selected for shorter lag times because of competition for nutrients (Monds et al., 2014), which may be coupled to reduced fitness in LB in long-term stationary phase (Philippe et al., 2009). Although cell size is highly correlated with lag time in some conditions (Monds et al., 2014), the mechanism connecting the two has not been elucidated. Nevertheless, our discovery that bigger cells are less likely to become regrowth deficient (Figure S2) suggests a direct connection between size and stationary/lag phase. A potential explanation consistent with our model for how larger cells maintain regrowth potential for longer in stationary phase is that the rate of damage accumulation is insensitive to cell size and hence larger cells are able to maintain the concentrations of proteins necessary for avoiding growth deficiency above a threshold. Delayed regrowth may thus act as a selective pressure against cells becoming too small (Shi et al., 2017). Regardless, our study underscores the dependence of stationary-phase physiology on how cells enter starvation and provides a road map for studying delayed regrowth across mutants and nutrient environments.

Our data extends a growing, common framework for growth-deficient cells, linking stationary phase to persistence and aging through protein aggregation, delayed regrowth, and oxygen dependence. The fact that aggregates are comparable in size to the diffraction limit in delayed-growth cells suggests that they form earlier in stationary phase but are too small to resolve. Until they reach a sufficiently large size (similar to that of polysomes), they may not end up segregated to the poles because of their ability to intercalate with the nucleoid (Coquel et al., 2013), and if they are not isolated at one pole, they may not be asymmetrically segregated upon division. The successful predictions of our deep learning model (Figure 3E) demonstrate the potential for development of a label-free tool that can utilize single-cell snapshots in imaging-enabled flow cytometers to sort populations based on regrowth potential, thus enabling analyses such as subpopulation-specific transcriptomics, proteomics, and metabolomics.

It is likely that most bacteria frequently enter stationary phase in their natural environment, particularly in the gut where periodic feeding leads to feast and famine. A screen of *Bacillus subtilis* CRISPRi knockdowns suggested that levels of essential genes have been determined by the need to survive stationary phase rather than for rapid growth (Peters et al., 2016) and adaptive mutations in budding yeast exhibit predictable tradeoffs between respiration and stationary phase (Li et al., 2019), indicating that stationary phase has proven a strong selective pressure. The heterogeneity of delayed regrowth (Figure 1) naturally presents the possibility for bet hedging, and similar heterogeneity could arise from other stresses that cause damage. Future investigation of species from environments such as the soil that constantly experience fluctuations in oxidative stress, compared with gut commensals (most of which are obligate anaerobes), may help to shed light on selection in stationary phase. Ultimately, stationary phase presents fascinating physiological challenges that provide a window into the fundamental mechanisms of growth.

### Limitations of the study

The low but nonzero level of persister formation during exponential growth (Wiuff et al., 2005) and the small growth defect of cells with older poles (Yu et al., 2019) suggests that damage is a small but ever-present challenge for bacteria, and in any event will introduce variability into studies of stationary-phase outgrowth (Simsek and Kim, 2019). Although the molecular triggers for aggregate formation have yet to be determined, our data suggest that respiration plays a major role (Figures 5B and S7F), which implies that facultative anaerobes may generally exhibit more growth heterogeneity in aerobic conditions. However, an alternate possibility is that the energy produced by electron transport chain activity contributes substantially to the development of protein aggregates by permitting ongoing unsuccessful attempts at protein synthesis under challenging conditions, distinct from direct oxidative damage caused by electron transport chain activity. Regardless, the ultimate consequences of cellular inability to remove aggregates are manifested in stationary phase, as evidenced by the aberrant morphologies and regrowth behaviors of Δ*dnaK* cells (Figures 4H–4J) despite growth that is similar to wild-type during log and early stationary phase (Figure 4G). Future studies that quantify and perturb *dnaK* regulation may help to shed light on aggregate dynamics.

## STAR★Methods

### Key resources table

**Table:** 

REAGENT or RESOURCE	SOURCE	IDENTIFIER
**Bacterial and virus strains**		

*Escherichia coli* MG1655	Huang laboratory strain collection	N/A
*E. coli* clinical isolates 1-4	(Galardini et al., 2017)	NILS1-4
*E. coli* MG1655 *mreB*^*A53T*^	Huang laboratory strain collection	N/A
*E. coli* MG1655 *PdnaK:gfp*	(Zaslaver et al., 2006)	N/A
*E. coli* MG1655 *PgroE:gfp*	(Zaslaver et al., 2006)	N/A
*E. coli* MG1655 *PrecA:gfp*	(Zaslaver et al., 2006)	N/A
*E. coli* MG1655 *PompA:gfp*	(Zaslaver et al., 2006)	N/A
*E. coli* MG1655 *Ppyk:gfp*	(Zaslaver et al., 2006)	N/A
*E. coli* MG1655 *PtnaC:gfp*	(Zaslaver et al., 2006)	N/A
*E. coli* MG1655 *PtufA:gfp*	(Zaslaver et al., 2006)	N/A
*E. coli* MG1655 *PrpsB:gfp*	(Zaslaver et al., 2006)	N/A
*E. coli* BW25113	Huang laboratory strain collection	N/A
*E. coli* BW25113 Δ*dnaK*	(Baba et al., 2006)	N/A

**Software and algorithms**		

μManager v. 1.4	(Edelstein et al., 2010)	N/A
Morphometrics	(Ursell et al., 2017)	N/A
Densenet-121	(Huang et al., 2017)	N/A
Custom code for mathematical modeling	https://bitbucket.org/kchuanglab/dormancy/src/master/	N/A

**Other**		

Millex-GP Syringe Filter Unit, 0.22 μm, polyethersulfone, 33 mm, gamma-sterilized	Millipore Sigma	SLGP033RS
Epoch 2 microplate spectrophotometer	Biotek Instruments	N/A
Eclipse Ti-E inverted fluorescence microscope with 100X (NA 1.40) oil-immersion objective	Nikon	N/A
Electron-multiplying CCD camera	Andor	DU885
sCMOS camera	Andor	Neo
Active-control environmental chamber	Haison Technology	N/A

### Resource availability

#### Lead contact

Further information and requests for resources and reagents should be directed to and will be fulfilled by the lead contact, Kerwyn Casey Huang (kchuang@stanford.edu).

#### Materials availability

This study did not generate new unique reagents.

### Experimental model and subject details

#### Bacterial strains

Strains used in this study, including *E. coli* MG1655 and BW25113 wild-type and Δ*dnaK* (Baba et al., 2006) strains, clinical isolates (Galardini et al., 2017), and fluorescent transcriptional reporter strains (Zaslaver et al., 2006), are listed in the Key Resources Table.

#### Strain culturing

All cultures were grown in 3 mL of filter-sterilized LB at 37°C in glass test tubes with constant shaking in aerobic conditions. Cultures were started at an OD at 600 nm (OD_600_) of 0.1 with cells from a 5-7 h culture inoculated from a frozen stock. Spent medium was isolated by spinning down (at 4000*g* for 5 min) and filtering a stationary-phase culture with a 0.22-μm polyethersulfone filter (Millex-GP SLGP033RS).

### Method details

#### Measurements of population growth

Growth curves were obtained using an Epoch 2 microplate spectrophotometer (Biotek Instruments, Vermont). The plate reader went through 15-min cycles of incubation at 37°C, shaking linearly for 145 s, and then absorbance measurements (wavelength 600 nm, 25 flashes, 2-ms settle between flashes).

#### Single-cell imaging

One microliter of cells was diluted 1:200 with fresh medium, spotted onto a pad of 1% agarose + LB, and imaged on a Nikon Eclipse Ti-E inverted fluorescence microscope with a 100X (NA 1.40) oil-immersion objective (Nikon Instruments). Phase-contrast and epifluorescence images were collected on a DU885 electron-multiplying CCD camera (Andor Technology) or a Neo sCMOS camera (Andor Technology) using μManager v. 1.4 (Edelstein et al., 2010). Cells were maintained at 37°C during imaging with an active-control environmental chamber (Haison Technology).

### Quantification and statistical analysis

#### Image analysis

The MATLAB (MathWorks, Natick, MA, USA) image processing code *Morphometrics* (Ursell et al., 2017) was used to segment cells and to identify cell outlines from phase-contrast microscopy images. A local coordinate system was generated for each cell outline using a method adapted from *MicrobeTracker* (Sliusarenko et al., 2011). Cell widths were calculated by averaging the distances between contour points perpendicular to the cell midline, excluding contour points within the poles and sites of septation. Cell length was calculated as the length of the midline from pole to pole. Cell area was used to calculate growth rate based on the formula 1/*A dA*/*dt*. Cell volume was estimated from cylindrical surfaces of revolution of local width measurements. Cellular dimensions (width/length/area/volume) were quantified by averaging single-cell results across a population. See figure legends for the number of cells analyzed (*n*) and error bar definitions.

#### Classification of subpopulations

A cell was classified as immediately growing if its median growth rate in the first 20 min of imaging was >0.004 min^−1^ and its growth rate eventually exceeded 0.01 min^−1^. Otherwise, a cell was classified as exhibiting delayed regrowth if its growth rate exceeded >0.005 min^−1^. The remaining cells were classified as nongrowing if their average growth rate at the conclusion of imaging was <0.003 min^−1^.

#### Detection of intracellular aggregates

Bright spots within cells in phase-contrast images were identified as aggregates if their maximum intensity was >5000 a.u. brighter than the median brightness of all pixels within the cell that were >2 pixels from the perimeter.

#### Deep learning classification of single-cell images

Densenet-121 (Huang et al., 2017) was trained on labeled images of cells cropped to 64x64 pixels. The training set included 2333 images of immediately growing cells, 788 images of delayed-growth cells, and 3225 images of nongrowing cells, from 9 imaging experiments. To augment the dataset, all images were mirrored and rotated 90, 180, and 270 degrees, resulting in 7 additional images per original image. Images were randomly partitioned between training, development, and test datasets with an 80%/10%/10% breakdown. All augmented images were placed in the same dataset as the original image. L2 regularization was added to Densenet-121 with a value of 0.0001. The parameters used for analysis of the test set were those from the 22^nd^ epoch of training, which was selected based on its performance with the development set.

#### Mathematical model and simulations

In our model (Figures 5 and S3), throughout stationary phase starting from ∼11 h after inoculation, cells accumulate damage from an initial value of 0, divide with a rate ρ, and transition between regrowth states (immediately growing, delayed regrowth, nongrowing over the period of observation, and non-viable) according to their damage levels. In a short time Δ*t* (which is sufficiently short to approximate a Poisson process), a cell with damage *A* undergoes the following: 1) damage increases to *A* + 1 with probability *δ*Δ*t*; 2) division occurs with probability ρΔt; and 3) if *A* > *A*_2_, the cell becomes non-viable with probability *γ*Δ*t*. During growth on fresh medium, cells are classified as immediately growing if *A* ≤ *A*_1_, delayed-growth if *A*_1_ < *A* ≤ *A*_2_, and nongrowing if *A* > *A*_2_.

Upon division, damage partitioning is asymmetric: one daughter receives all damage, while the other daughter is returned to an undamaged state. Division always coincides with the removal of a randomly selected cell to maintain population size. Simulations based on this model were carried out using the Gillespie Algorithm (Gillespie, 1977) with 1,000 cells; note that Gillespie Algorithm effectively implements a time step Δ*t* that is small compared to any rate constant.

#### Analytical expressions for the proportion of immediately growing and delayed regrowth cells

To derive analytical expressions for the steady states of our model, we specify a continuum version of our stochastic model of damage-induced delayed regrowth that has the same steady states as the stochastic model. In the continuum model, *g* is the fraction of immediately growing cells with aggregates *A* < *A*_1_, r is the fraction of delayed cells with *A*_1_ < *A* < *A*_2_, $\bar{g}$ is the fraction of cells with *A* > *A*_2_ that do not grow but still divide, and e is the fraction of cells that cannot divide or that are dead but not lysed. Average deterministic equations corresponding to the stochastic model are:

$$\frac{dg}{dt}=\frac{\delta }{A_{2}}(\frac{\rho A_{2}}{\delta }\left(1-e\right)-(\frac{A_{2}}{A_{1}}+\frac{\rho A_{2}}{\delta }\left(1-e\right))g)$$

$$\frac{dr}{dt}=\frac{\delta }{A_{2}}(\frac{A_{2}}{A_{1}}g-\;(\frac{1}{1-A_{1}/A_{2}}+\frac{\rho A_{2}}{\delta }\left(1-e\right))r)$$

$$\frac{d\bar{g}}{dt}=\frac{\delta }{A_{2}}(\frac{1}{1-A_{1}/A_{2}}r-\;(\frac{\gamma A_{2}}{\delta }+\frac{\rho A_{2}}{\delta }\left(1-e\right))\bar{g})$$

$$\frac{de}{dt}=\frac{\delta }{A_{2}}\;\left(\frac{\gamma A_{2}}{\delta }\bar{g}-\;\frac{\rho A_{2}}{\delta }\left(1-e\right)e\right)$$

where we select *δ* / *A*_2_ to govern the timescale, and other governing parameters are *A*_1_ / *A*_2_, $\rho A_{2}/\delta$, and *γ A*_2_ / *δ*. For model (b) with damage-induced delayed regrowth (*δ* > 0) and division (ρ>0) but no death (*γ* = 0, *e* = 0), at steady state $g=\rho A_{1}/\delta /(1+\rho A_{1}/\delta )\;$ and $g/r=\frac{1}{A_{2}/A_{1}-1}+\rho A_{1}/\delta$. For model (c) including death (*γ* > 0), all cells eventually die (so eventually *e* = 1) when $\rho^{-1}>\;A_{2}\delta^{-1}+\gamma^{-1}$, which is the case for the parameters in Figure 5D. Figure S3B shows how steady states but not earlier dynamics of the stochastic model match the continuum model.

#### Dynamics are similar when the division rate decreases linearly with damage

Here, instead of a constant division rate ρ and a constant rate *γ* of switching to non-division (or death without lysis) when *A* ≥ *A*_2_ in model (c) (Figure 5A), division rate decreases with damage according to $\frac{\rho A_{2}}{\delta }=\bar{\frac{\rho A_{2}}{\delta }}\left(1+\alpha \left(0.5-\frac{A}{A_{2}}\right)\right)$ and $\rho A_{2}/\delta =0$ if *A* ≥ *A*_2_ (*α*^-1^ + 0.5). Thus, when *A* = *A*_2_ (*α*^-1^ + 0.5), cells enter a non-dividing state. If *α* = 0, the non-dividing state is never reached, whereas if *α* = 2, division halts when *A* = *A*_2_ coincidentally with the cell entering the nongrowing state.

## Data Availability

All data used in this manuscript are growth curves and microscopy images. All data are available upon request from the corresponding author, and code is available at https://bitbucket.org/kchuanglab/dormancy/src/master/. Any additional information required to reanalyze the data reported in this paper is available from the lead contact upon request.
