# Supplementary material for: Bacterial respiration during stationary phase induces intracellular damage that leads to delayed regrowth
Source: iScience. 2022 Jan 15;25(3):103765. doi: 10.1016/j.isci.2022.103765 (PMC8858994; doi:10.1016/j.isci.2022.103765)
Supplement: Document S1. Figures S1–S7 [file mmc1.pdf]

iScience, Volume *25*

Supplemental information

Bacterial respiration during stationary phase induces intracellular damage that leads to delayed regrowth

Spencer Cesar, Lisa Willis, and Kerwyn Casey Huang

Supplemental Figure Legends

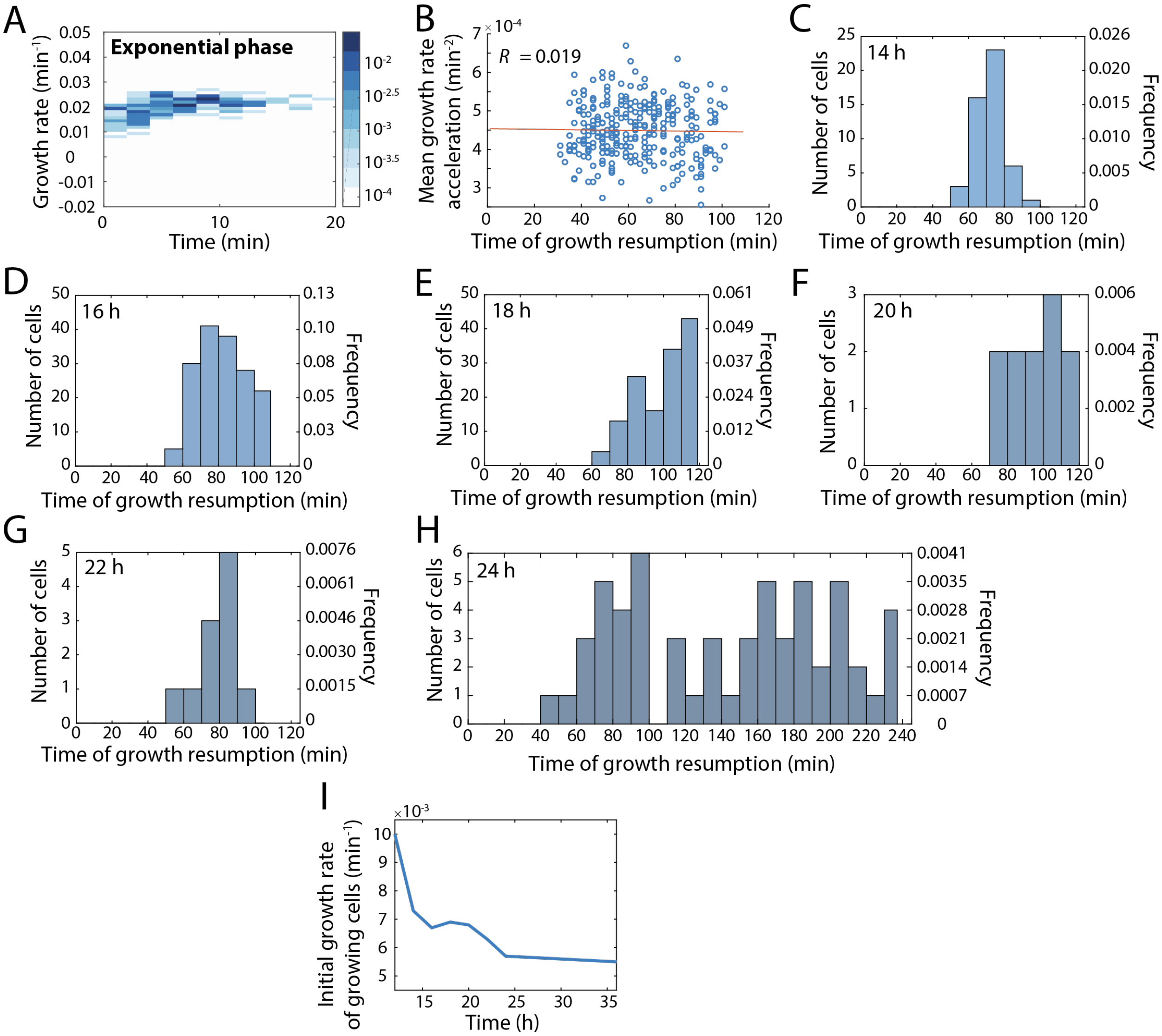

Figure S1: The time at which cells resume growth increases with increasing time in stationary phase, and immediately growing cells start with a lower growth rate. Related to Figure 1.

A) Heatmap of the distribution of instantaneous growth rates over time for exponential-phase cells placed on agarose pads made with fresh LB. Cell area

was used to calculate growth rate using the formula $1/A\ dA/dt$. n>200 cells for each time point.

B) The average acceleration in growth rate (computed for each cell as the slope of a line fitted to data from 18 min before to 8 min after achieving a growth rate of 0.02 min^{-1}) was uncorrelated with the time at which cells from a 16-h culture resumed growth. n>200 cells.

C-H) The time at which cells resumed growth increased with increasing culture age. Dashed lines are means. The frequency was measured including nongrowing cells.

I) The initial growth rate of immediately growing cells decreased as a function of culture age. Numbers of cells are the same as for each distribution in (C-H).

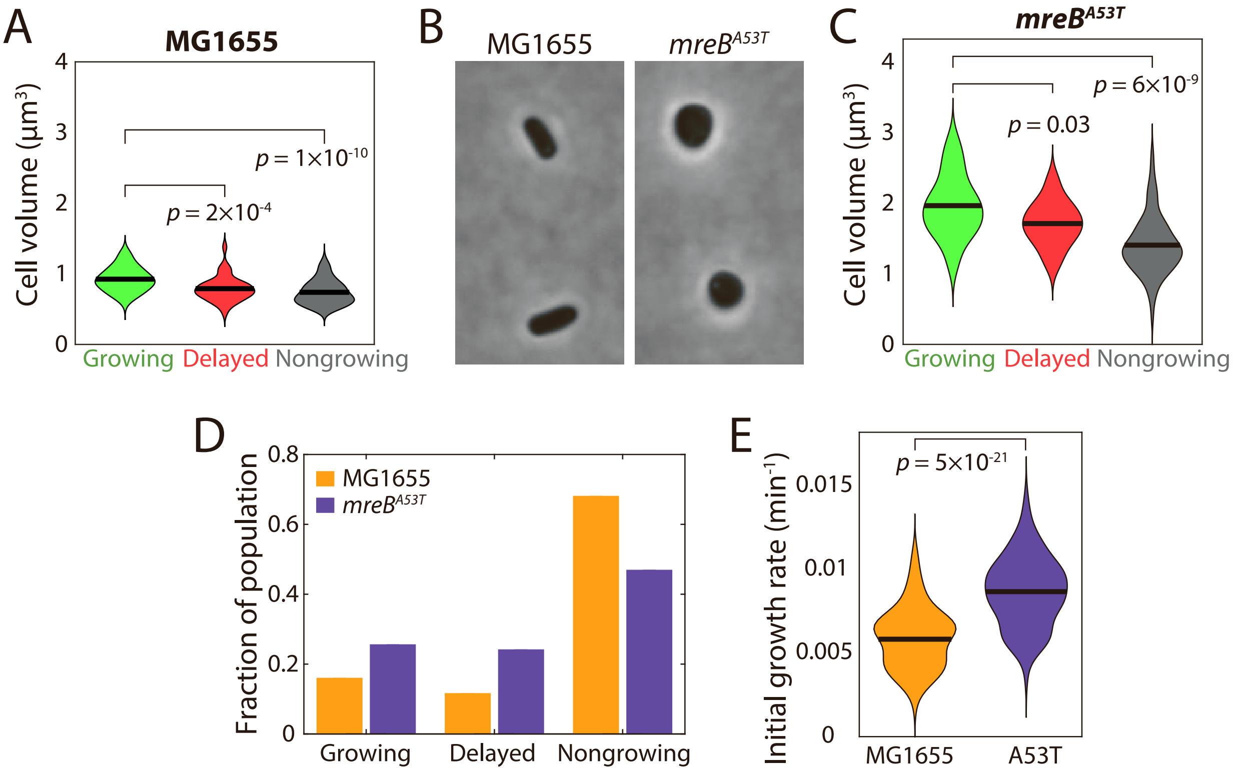

Figure S2: Cells with larger volume are enriched in the resuscitating populations, and mutants with increased volume have higher growth potential during emergence from stationary phase. Related to Figure 1.

A) Delayed-growth cells from a 20-h culture were significantly smaller in the first frame of imaging than immediately growing cells, and nongrowing cells were smaller yet. Thick horizontal lines are mean values. *n*>100 cells for each subpopulation.

B) *mreB*^{A53T} cells from a 20-h culture were wider and larger in volume than wild-type MG1655 cells.

C) *mreB*^{A53T} cells displayed a similar qualitative relationship between cell size and regrowth behavior as wild-type cells in (A), although *mreB*^{A53T} cells were larger overall. Thick horizontal lines are mean values. n>100 cells for each subpopulation.

D) The *mreB*^{A53T} population in (C) had a larger fraction of immediately growing and delayed regrowth cells and a smaller fraction of nongrowing cells compared with wild type (A). Fractions were calculated using n>400 cells.

E) The initial growth rates in the first 8 min of time-lapse imaging of immediately growing *mreB*^{A53T} cells were higher than those of wild-type cells, indicating that larger cells were generally more capable of stationary-phase exit. Thick horizontal lines are mean values. n>100 cells for each strain.

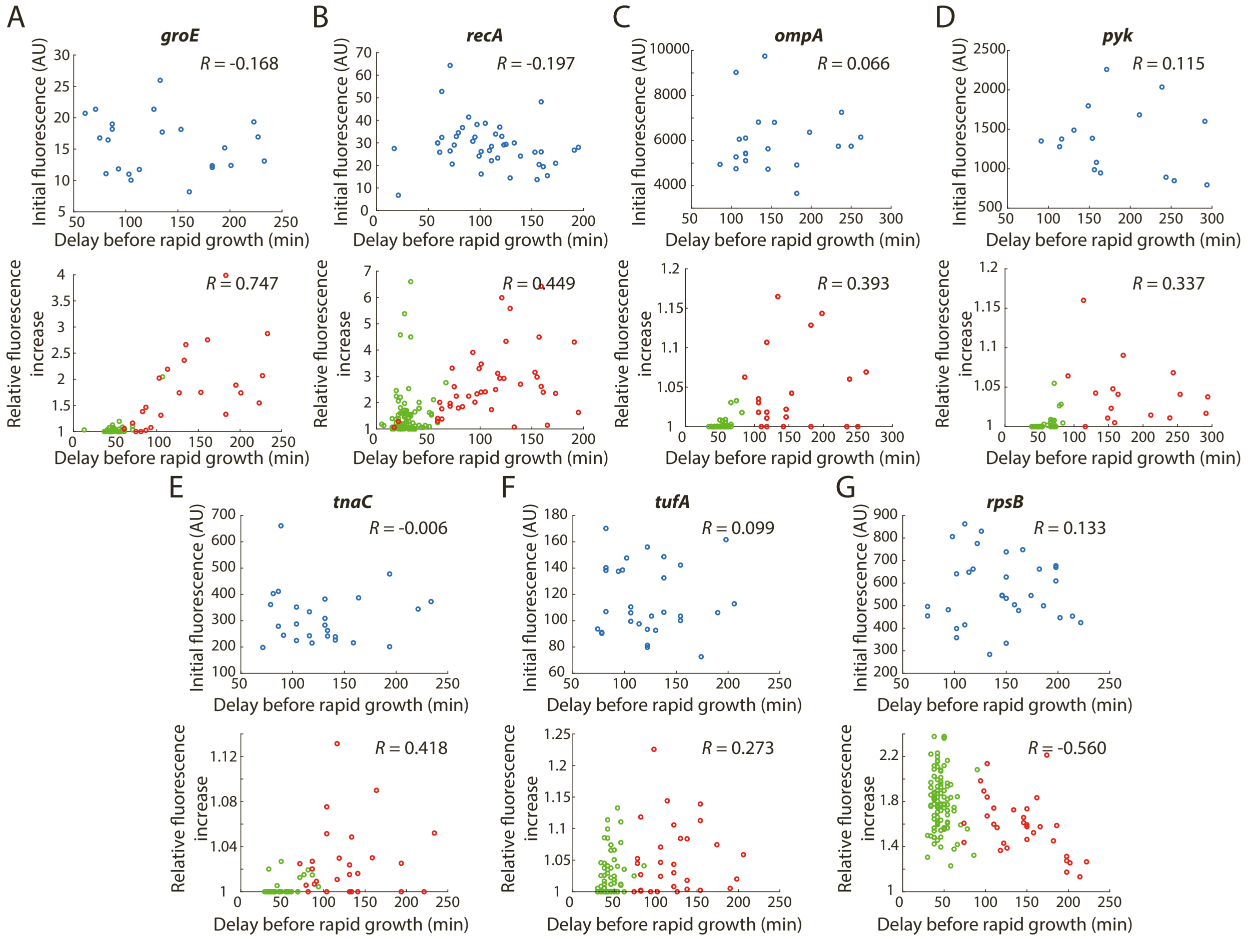

Figure S3: Chaperones are upregulated in delayed regrowth cells, but not genes related to metabolism or translation. Related to Figure 4.

A) Similar to *dnaK* (Fig. 4B,E), initial GFP fluorescence concentration from the promoter of *groE* was uncorrelated with the delay before rapid growth (defined as the time to reach growth rate 0.02 min^{-1}) (top), but the maximum fluorescence concentration during time-lapse imaging on fresh medium relative to the initial fluorescence concentration was correlated with the delay before rapid growth (bottom). *n*≥23 cells. These data support the hypothesis that delayed regrowth is due to accumulated protein damage.

B-G) By contrast, for several non-damage-protein-related promoters including *recA* (B, $n \geq 43$ cells) *ompA* (C, $n \geq 21$ cells), *pyk* (D, $n \geq 16$ cells), *tnaC* (E, $n \geq 26$ cells), *tufA* (F, $n \geq 30$ cells), and *rpsB* (G, $n \geq 33$ cells), GFP concentration increases were uncorrelated with the delay until rapid growth.

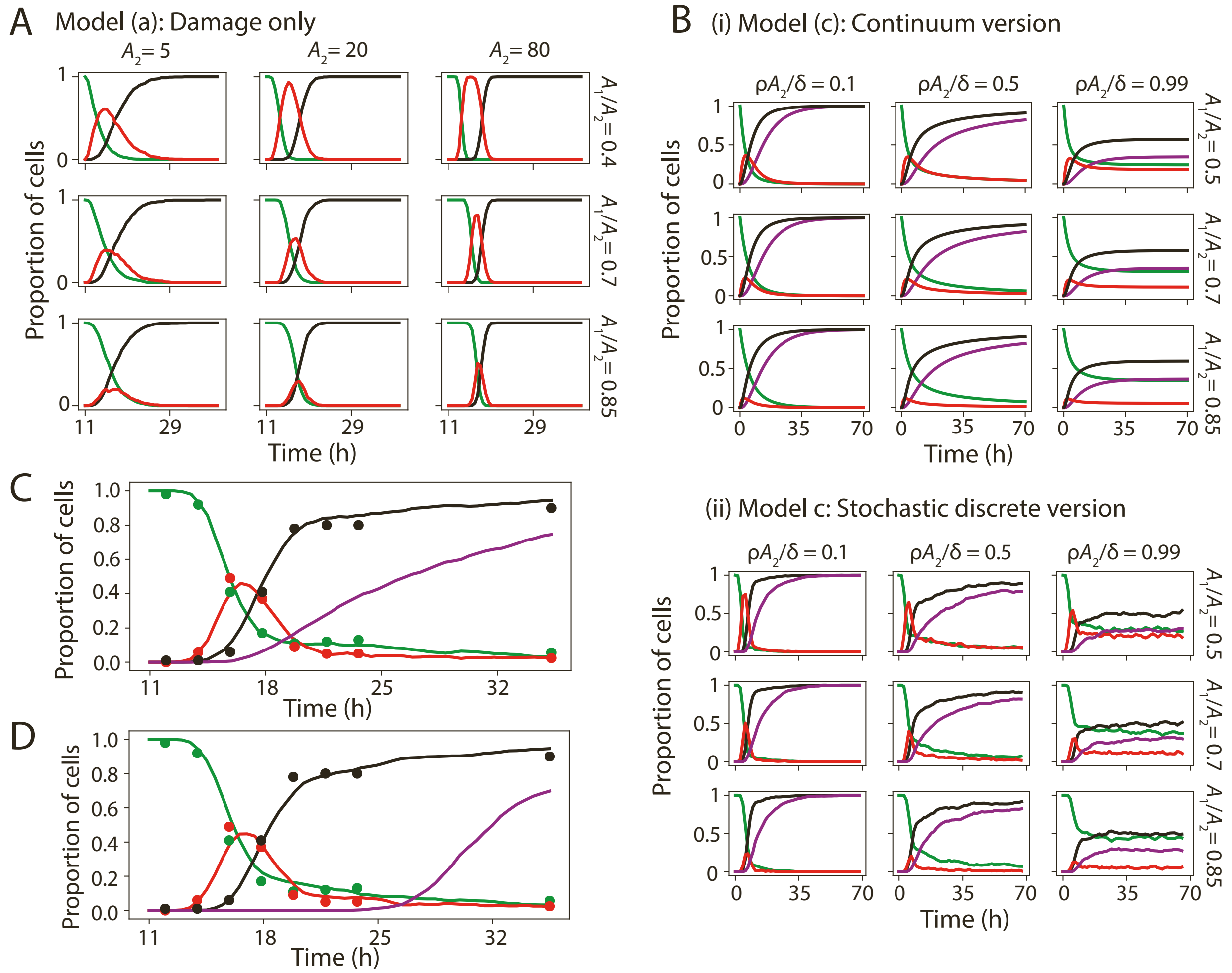

Figure S4: Mathematical model makes similar predictions if a threshold intracellular concentration of damage triggers delayed regrowth, or if division rate declines with damage. Related to Figure 5.

(A)Simulations of model (a) (Fig. 5A) with damage-induced delayed regrowth but no division or death recapitulated regrowth statistics up to, but not beyond, ~20 h after inoculation when $\frac{A_1}{\delta} \approx 5$ h, $\frac{A_2}{\delta} \approx 7$ h, so $\frac{A_1}{A_2} = 0.7$, and $A_2 = 20$. In all panels, immediately growing cells are green, delayed-regrowth cells are red, and nongrowing cells are black. A_2 tuned the time of the peak in the delayed-

regrowth fraction, while both the peak and duration of the delayed regrowth fraction were tuned by A_1/A_2.

(B) Steady states but not the early dynamics of the continuum model (i) (Supplementary Text) agreed with the stochastic model (ii), unless $A_2 \approx 1$ or $A_1 \approx A_2$ in which case discretization effects were strong (compare bottom right panels for (i) and (ii)). Parameters are $\frac{A_2}{\delta} \approx 7$ h, $A_2 = 20$ (for (i)), and $\frac{\gamma A_2}{\delta} = 0.8$, while $\frac{A_1}{A_2}$ and $\frac{\rho A_2}{\delta}$ vary. Purple indicates the subpopulation of non-dividing or dead cells.

(C) The dynamics of model (c) are similar if a threshold intracellular concentration of damage triggers delayed regrowth, as opposed to a threshold absolute level of damage. Here, A now represents damage concentration, which doubles in the daughter cell that inherits all damage upon division. Other parameter definitions are unchanged. Parameters $\frac{A_1}{\delta} \approx 5$ h, $\frac{A_2}{\delta} \approx 7$ h, so $\frac{A_1}{A_2} = 0.7$, $A_2 = 20$, $\frac{\rho A_2}{\delta} = 0.16$, and $\frac{\gamma A_2}{\delta} = 0.8$, as for Fig. 5D, produce an excellent fit. Solid lines correspond to the model and circles correspond to data.

(D) The dynamics of model (c) are similar if instead of a death rate $\gamma > 0$ when $A > A_2$, the division rate declines linearly to 0 with increased damage (Supplementary Text). Parameters $\frac{A_1}{\delta} \approx 5$ h, $\frac{A_2}{\delta} \approx 7$ h, so $\frac{A_1}{A_2} = 0.7$, $A_2 = 20$, $\frac{\overline{\rho A_2}}{\delta} = 0.25$, and $\alpha = 0.45$ produce an excellent fit with a slight lag in cell death (purple). Solid lines correspond to the model and circles correspond to data.

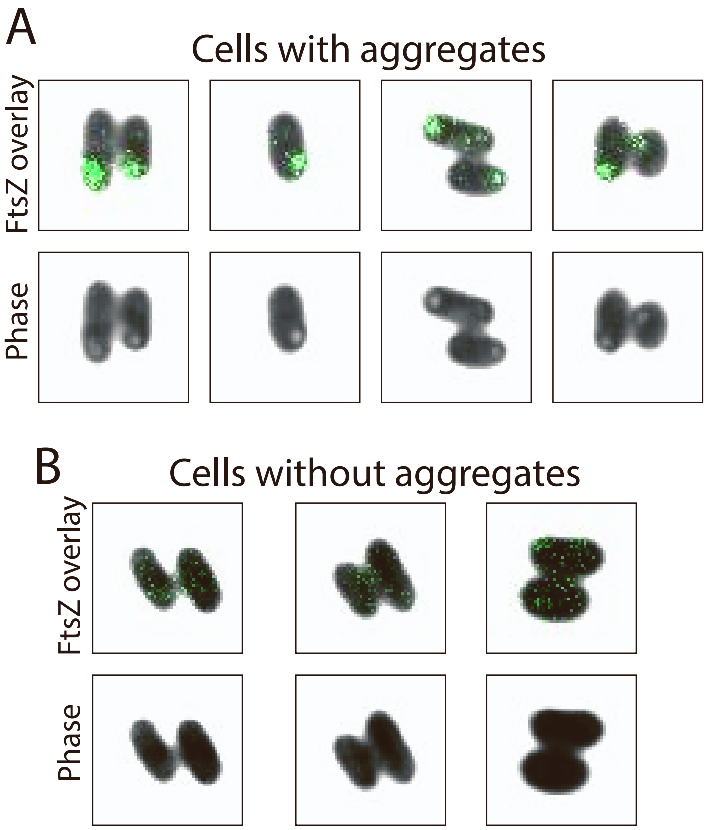

Figure S5: FtsZ is sequestered within aggregates at the cell poles in stationary phase. Related to Figure 5.

B) Top: overlay of FtsZ-msfGFP fluorescence with phase-contrast images of cells with aggregates. Bottom: phase-contrast images showing bright foci at the same intracellular locations as the FtsZ punctae. Similar behavior was observed in n>100 cells.

C) Top: overlay of FtsZ-msfGFP fluorescence with phase-contrast images of cells without aggregates showing diffuse fluorescence in cells without aggregates. Bottom: phase-contrast images. Similar behavior was observed in n>100 cells.

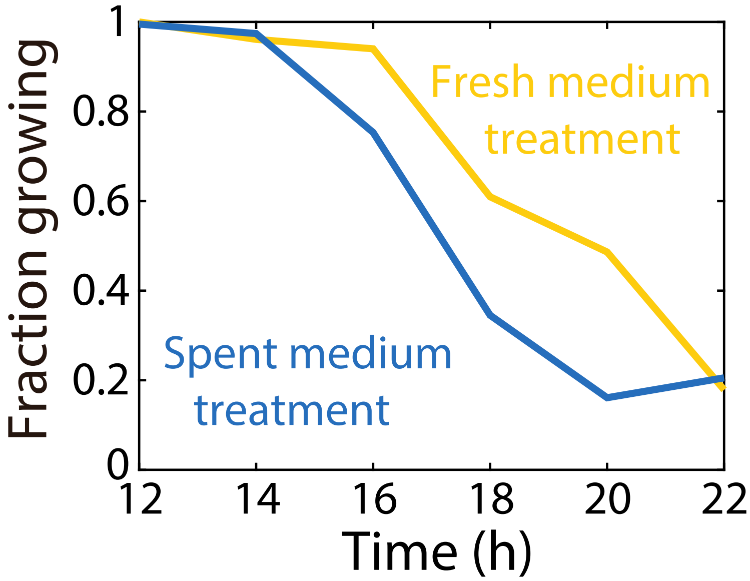

Figure S6: A pulse of fresh medium delays the onset of delayed regrowth. Related to Figure 5.

Resuspension of cells from a 12-h culture in fresh LB for 20 min (yellow) led to a ~2-h delay in the onset of delayed regrowth following resuspension in spent supernatant relative to a control that did not experience the pulse of fresh medium, indicating that the cause of delayed regrowth can be reversed. Fractions were computed with *n*>100 cells.

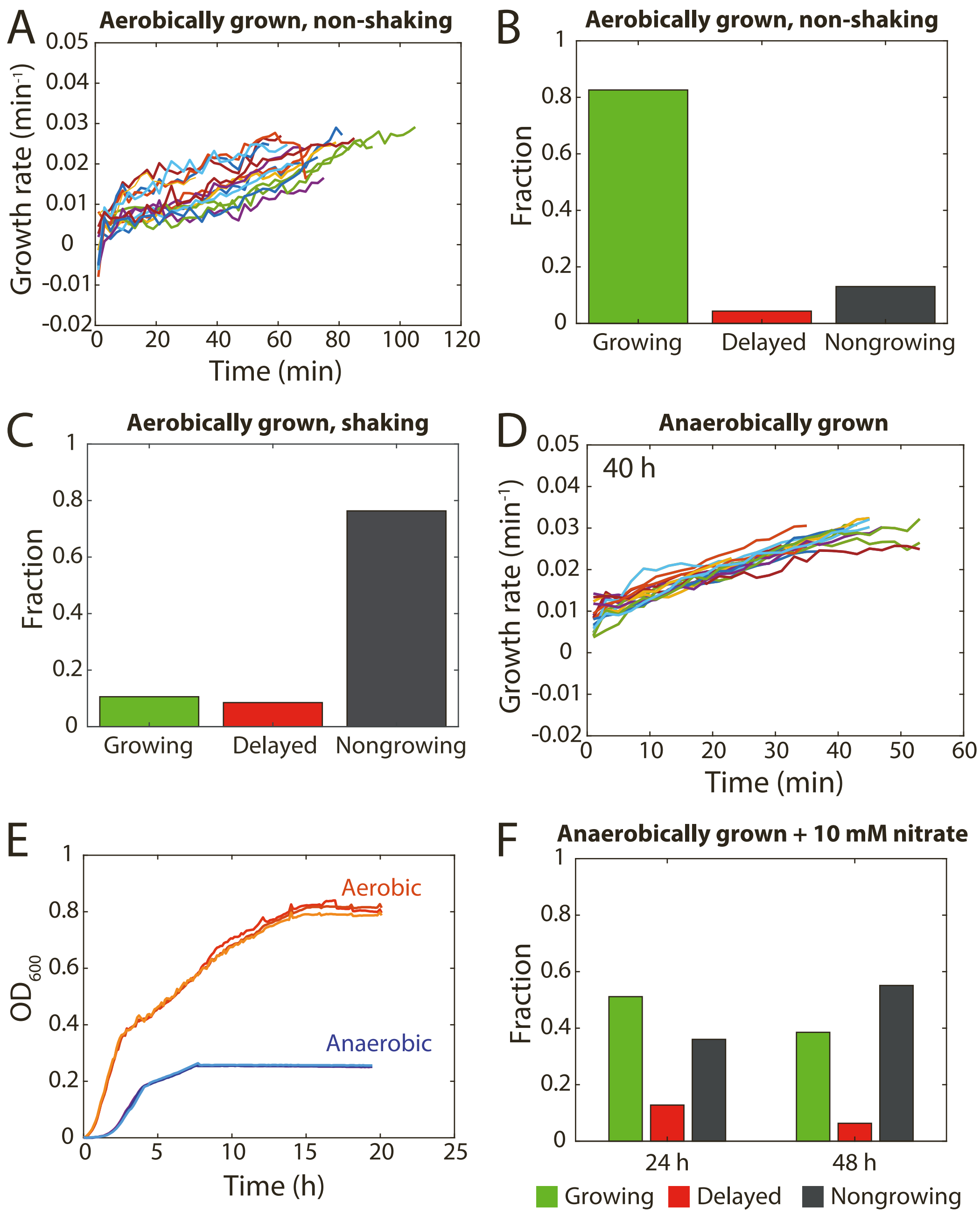

Figure S7: Anaerobically grown cells resume growth immediately coming from stationary phase unless nitrate is available for respiration during stationary phase. Related to Figure 6.

A) After 20 h of growth in non-shaking conditions, most cells were able to grow immediately upon exposure to fresh medium. 14 representative cells are shown (similar behavior was observed in *n*>100 cells).

B,C) Fractions of each subpopulation after 20 h of aerobic growth in non-shaking conditions show that most cells can grow immediately (B), as compared with the large proportion of nongrowing cells in shaking conditions (C, reproduced from Figure 1K). Fractions were computed from *n*>100 cells.

D) Cells grown in an anerobic chamber for 40 h immediately resumed growth on fresh LB agarose pads, suggesting oxidative stress is a primary cause of delayed regrowth. 34 representative cells are shown (similar behavior was observed in *n*>100 cells).

E) *E. coli* saturates at a lower yield in LB when grown in anaerobic versus aerobic conditions. Shown are three technical replicate growth curves.

F) After 24 h of anaerobic growth in LB supplemented with 10 mM nitrate, almost half of the population was delayed in regrowth or nongrowing. After 48 h, the nongrowing population expanded at the expense of the fractions of both immediately growing and cells with delayed regrowth. Fractions were computed from *n*>100 cells.
